# Characterization of Natural Organic Matter in Conventional Water Treatment Processes and Evaluation of THM Formation with Chlorine

**DOI:** 10.1155/2014/703173

**Published:** 2014-01-16

**Authors:** Kadir Özdemır

**Affiliations:** Department of Environmental Engineering, Bülent Ecevit University, Incivez, 67100 Zonguldak, Turkey

## Abstract

This study investigates the fractions of natural organic matter (NOM) and trihalomethane (THM) formation after chlorination in samples of raw water and the outputs from ozonation, coagulation-flocculation, and conventional filtration treatment units. All the water samples are passed through various ultrafiltration (UF) membranes. UF membranes with different molecular size ranges based on apparent molecular weight (AMW), such as 1000, 3000, 10,000, and 30,000 Daltons (Da), are commonly used. The NOM fraction with AMW < 1000 Da (1 K) is the dominant fraction within all the fractionated water samples. Its maximum percentage is 85.86% after the filtration process and the minimum percentage is 65.01% in raw water samples. The total THM (TTHM) yield coefficients range from 22.5 to 42 **μ**g-TTHM/mg-DOC in all fractionated samples, which is related to their specific ultraviolet Absorbance (SUVA) levels. As the molecular weight of the fractions decreased, the TTHM yield coefficients increased. The NOM fractions with AMW values less than 1 K had lower SUVA values (<3 L/mg*·*m) for all treatment stages and also they had higher yield of TTHM per unit of DOC. The NOM fraction with AMW < 1 K for chlorinated raw water samples has the highest yield coefficient (42 **μ**g-TTHM/mg-DOC).

## 1. Introduction

Chlorination has been used for disinfection to eradicate pathogenic microorganisms from drinking water [1–3]. Nevertheless, the formation of chlorinated byproducts such as trihalomethanes (THMs) and haloacetic acids (HAAs) is related to reactions between chlorine and natural organic matter (NOM) [4, 5]. Furthermore, several studies have noted that disinfection byproducts (DBPs) have been generated as a result of the chlorination of organic matters in water [6]. Of the DBPs formed in chlorinated water, THMs represent a substantially greater fraction. Also, these products have adverse health effects on human beings and are considered potentially carcinogenic water [7, 8]. Therefore, the major international regulatory agencies such as the United States Environmental Protection Agency (USEPA) and European Union (EC) have developed a number of regulations for DBPs like THMs [3, 9]. The USEPA has set maximum contaminant levels for THMs (chloroform, bromodichloromethane, dibromochloromethane, and bromoform) of 80 *μ*g/L. On the other hand, the EC regulation limit for total THM concentration in drinking water is 100 *μ*g/L [10]. In Turkey, the THM limit is also 100 *μ*g/L [11].

NOM has been recognized as the most important source of DBPs precursors [12, 13]. The characteristics of NOM are of great significance in the water treatment processes [14]. Moreover, not only the chemical but also the physical properties of NOM play major roles in conventional treatment process such as ozonation, coagulation, filtration, and disinfection [15]. Therefore, the most common fractionation techniques known as resin adsorption process [16, 17] and ultrafiltration have been applied successfully for the characterization of NOM in the past years [18].

Ultrafiltration (UF) is a simple fractionation technique used to separate NOM into different molecular size ranges based on apparent molecular weight (AMW) [19, 20]. UF membranes have different molecular weight cut-offs (MWCOs); values such as 1000, 3000, 5000, 10000, and 30000 Daltons (Da) are commonly used [21]. Meanwhile, one of the most significant advantages of the UF techniques is that there is no requirement for chemical reagents to be added to the samples [22]. The determination of NOM fractions in water samples, based on dissolved organic carbon (DOC) mass balance, is necessary to better represent the real composition of the NOM. It is reported that the molecular weight of most dissolved organic matter (DOM) in the Pearl River water sample was <500 Da and its percentage reached 58% [23]. Many investigators have studied the molecular size distribution of NOM and the consequent DBPs reactivities after chlorination [24, 25].

The main objectives of this research were (i) to determine THMs formation from different molecular weight fractions of NOM in the disinfection process using chlorine and (ii) identify the main precursor of the disinfection byproducts among the different fractions of NOM.

## 2. Materials and Methods

### 2.1. Sample Collection

Raw and processed water samples were collected from the Kağıthane drinking water treatment plant (KWTP) in İstanbul, within a summer season in 2010. Raw water is transferred from Terkos Lake to KWTP, in İstanbul, Turkey. Terkos Lake is one of the most important water reservoirs in İstanbul and provides a maximum of 700,000 m^3^/day of raw water to KWTP. KWTP is a common conventional treatment plant including prechlorination, ozonation, coagulation-flocculation, and filtration process units. The oxidation of NOM in raw water is performed with chlorine and ozone gases. The applied optimal ozone and chlorine doses in raw water were nearly 2.5 mg/L and 3 mg/L, respectively. Raw waters were coagulated using alum, for which the average applied dose was 60 mg/L. Finally, coagulated waters were passed through the rapid sand filters and then the filtered waters were disinfected with chlorine. The physicochemical characteristics of raw water quality parameters are shown in Table 1. Raw and processed water samples were collected in 1 L glass bottles. They were cleaned with deionized ultrapure water (DIUF) on the sampling day. Samples were rapidly shipped to the KWTP laboratory. Then, all water samples were passed through the 0.45 *μ*m membrane filter papers within 24 h and stored in a refrigerator at +4°C to retard microbial activity prior to use.

### 2.2. Molecular Size Fractionation of NOM by Ultrafiltration

**Figure 1:**
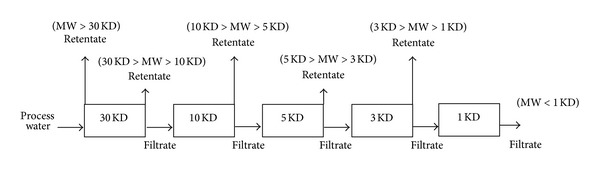
UF serial processing scheme.

The ultrafiltration (UF) process was used to fractionate the molecular size of NOM in water samples taken from each treatment stage: raw water, ozonation, coagulation-flocculation, and filtration. In this study, UF was carried out by using a stirred UF cell (Millipore 8200) with YM disc membrane (Amicon, USA) and molecular weight cut-off (MWCOs) membranes including 1, 3, 5, 10, and 30 K. The scheme of the UF process is illustrated in Figure 1. Each of the water samples was ultrafiltrated sequentially at 30, 10, 5, 3, and 1 K. Prior to the fractionation process, the apparatus was cleaned according to the procedure of Zhao et al. [23] as follows. Firstly, membranes were soaked several times (not less than three times) with DIUF to remove glycerin which was added by the producers to the membrane to avoid drying in shipment. After having been soaked, the membrane was placed in the UF cell pressurized with nitrogen gas in the range from 20 to 35 kPa. DIUF was then passed through the UF cell with the membranes installed to remove any organic impurities.

### 2.3. Chlorination Procedure

Fractionated water samples were chlorinated following the procedure described in Standard Methods 5710 B [26]. The chlorination process was conducted for a given chlorine dosage (10 mg/L), fixed pH (pH 7), and room temperature (20°C). The chlorinated NOM fractions for each treatment unit process were transferred to 100 mL amber glass bottles with screw caps and TFE-faced septa. After chlorination, the fractionated samples were incubated at 20°C for the desired contact time (24 h). At the end of the reaction period, a quenching agent (sodium sulphite solution) was added to each of the chlorinated water samples for the analysis of THM formation.

### 2.4. Analytical Procedure

After all the water samples had been fractionated by the UF process, they were analyzed for DOC, UV absorbance, and THM measurements. DOC analyses were performed with a Shimadzu TOC-5000 Analyzer equipped with autosampler, using the persulphate oxidation method as described in Standard Methods 3510 C. A UV −1608 Shimadzu spectrophotometer was used for measurements of UV absorbance at 254 nm wavelength. Specific UV absorbance (SUVA) was calculated as the UV_254_ absorbance divided by the DOC concentrations. THM analyses were conducted as liquid-liquid extraction (LLE) with pentane. THM samples were pipetted to 40 mL EPA vials, after that 3 mL of pentane was added to each vial. The samples were shaken vigorously by hand from one to minutes to ensure phase separation. The pentane extract from each vial was measured by an Agilent Gas Chromatography (GC) instrument equipped with a microelectron capture detector (GC-*μ*ECD), autosampler, and a fitted capillary column, (J&W Science DB-1), 30 m∗0.32 mi.d. ∗ 1 *μ*m film thickness. The sum of four THM compounds (chloroform, dichlorobromomethane, dibromochloromethane and bromoform) was reported as total THM (TTHM), in *μ*g/L.

## 3. Results and Discussion

### 3.1. Mass Distribution of the Fractioned NOM

UF plays a significant role in conventional drinking water treatment processes. Further, the UF technique is widely used for the determination of molecular weight distributions of NOM in water treatment and membrane technologies. This investigation includes two main goals. The first one is to fractionate water samples containing NOM taken from the KWTP processing units and establish the carbon mass balance of the UF processes according to the DOC measurements. The other goal is to determine the formation potential of THMs produced by the chlorination of the different NOM fractions in each water treatment stage. Prior to UF processes, a mass balance should be performed for each NOM fraction as regards DOC analysis. In other words, any loss or contamination was evaluated with mass balance based on the DOC concentrations of NOM fractions. The results of the mass balance calculations for each NOM fraction which are related to the KWTP stages (raw water, ozonation, coagulation-flocculation, and filtration) are given in Table 2.

**Figure 2:**
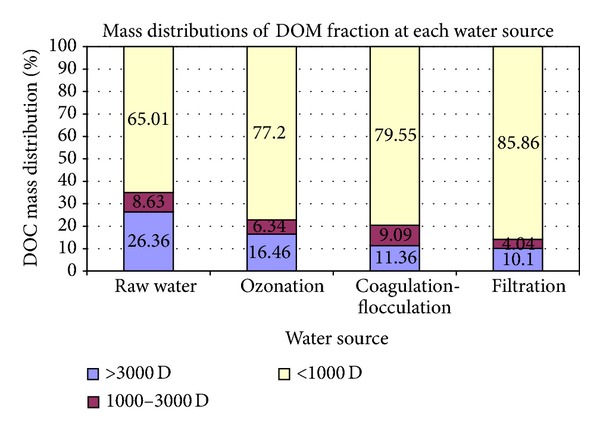
Fraction mass distribution of water treatment processes.

According to Table 2, the analyzed DOC concentration of each NOM fraction in the different treatment units is presented in the left column in units of mg/L. On the other hand, the volume of water samples as unit of mg/L was given in the middle column and the mass of DOC for each NOM fraction was calculated in the right hand column as unit of mg. The distribution of DOC concentrations for different fractions was determined by the DOC mass value of each NOM fraction divided by the sum of DOC mass value of all fractions. All the same, the distributions of DOC for NOM fractions based on the calculation of mass balance and DOC recoveries are shown in the last column of Table 2 as a percentage (%). The results of DOC recovery were good: DOC recoveries including those in raw water, ozonation, coagulation-flocculation, and filtration process outputs were calculated as (100 ± 10.21%), (100 ± 6.2%), (100 ± 3.53%), and (100 ± 7.41%), respectively. Gang et al. [21] have reported that mass balance on DOC and UV_254_ recoveries is better than (96 ± 3.2%) for raw water samples. The distribution of NOM fractions for each water sample with regard to DOC concentrations is presented in Figure 2.

According to Figure 2, the AMW < 1 K fraction of the organic matter was predominant. Moreover, the percentage of its DOC content changed nearly between 65% and 85% within the four treatment stages. On the other hand, the ratio of the NOM fractions with AMW > 3 K and 1–3 K were between 16% and 6%, as compared with the fraction of AMW < 1 K for all water samples (Table 2). In this study, the outcomes of DOC concentration determination which were related to the fractions of AMW > 30 K, 30–10 K, 10–5 K, and 5–3 K were not reported because the DOC measurements of these fractions were <0.1 mg/L in all water samples and therefore not reliable. Our results confirm the findings by Wei et al. [15] that the fraction of AMW less than 1 K comprises the largest part of the DOC content in all four raw water samples.

Zhao et al. [23] reported that the molecular weight of most NOM in water samples collected from the Pearl River was less than 0.5 K. As shown in Figure 2, the ratio of the organic fraction with AMW less than 1 K was observed to show a quite marked difference between raw water compared to the filtration process water. For instance, although its ratio was 65.5% prior to ozonation, it increased to 77.2% during the ozonation stage. This result also revealed that the ratio of NOM fractions less than 1 K increases slightly as the ozonation process leads to the partial oxidation of NOM.

### 3.2. The Distribution of Various NOM Fractions on DOC, UV_**254**_, and THM Formation

**Figure 3:**
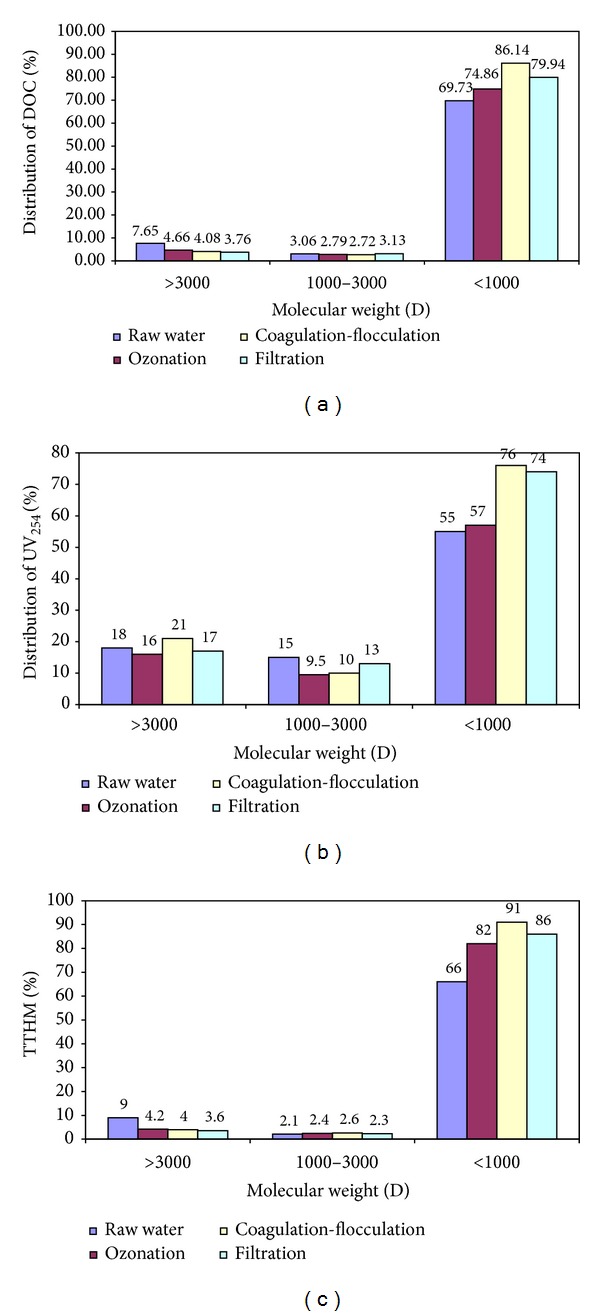
Percentages of distributions for (a) DOC and NOM fractions, (b) UV_254_ and NOM fractions, and (c) TTHM and NOM fractions.

In this part of the study, variations on the values of DOC and UV_254_, with respect to AMW distributions from water samples at different treatment steps in KWTP, are presented in Figure 3. Checking the DOC and UV_254_ values in Figure 3, the biggest contribution was from the fraction with AMW less than 1 K within all water treatment processes. The organic matter with MMW less than 1 K occupied about 70–75% of the DOC concentration in the raw water and ozonation steps and this ratio reached about 80–86% in the coagulation and filtration processes, respectively. On the contrary, the fraction with AMW greater than 3 K and the fraction with AMW 1–3 K had only about 4–8% and 2-3% of the total DOC for all treatment stages, respectively (Figure 2). A similar trend was observed for UV_254_ values; for example, the NOM fraction with AMW < 1 K represented as 55–60% of UV_254_ values in the raw water and ozonation stages and was determined to be about 74% and 76% in coagulation and filtration processes, respectively. Nonetheless, the UV_254_ percentages of the other NOM fractions (AMW > 3 K and 1 K−3 K) ranged from 10% to 20% at all treatment units (Figure 2). It was observed that there was little difference between DOC and UV_254_ values of the NOM fraction with AMW < 1 K for all water samples. This finding can be expressed by the fact that while DOC measurements give us information about total NOM concentration, UV_254_ readings shows only the concentrations of humic substances in water. On the other hand, the DOC and UV_254_ values of the other NOM fractions are very low; for example, the contribution of these fractions (AMW > 3 K and 3 K−1 K) was around maximum of 20% of DOC and UV_254_ values in all water samples (Figure 3).

As compared to all the water samples taken from each treatment unit in KWTP, the highest DOC and UV_254_ values were analyzed at the fraction of AMW < 1 K among the other fractions in raw water samples, as 4.1 mg/L and 0.06 cm^−1^. On the other hand, Figure 3 shows the percentage of TTHM formation within the reaction time of 24 h (TTHM_24 h_) for each chlorinated NOM fraction. Comparing the TTHM formation for all NOM fractions in the KWTP processing units, about 65–90% TTHM is generated from chlorinating the fraction of AMW < 1 K in all water samples. As its value was 66% of the total in raw water, it reached about 90% of that in filtrated water. Besides, the TTHM percentages of the other fractions varied from 9% to 2% at all treatment steps. These findings therefore show that the low-molecular-weight fractions (<1 K), defined as hydrophilic compounds, play a greater role in the formation of THMs. Besides, Zhao et al. [23] suggested that the low-molecular-weight 0.5–1 K fraction was the major precursor of THM formation for effluent of each of the four treatment processes in a conventional drinking water plant in Guangzhou.

### 3.3. SUVA and THM Reactivity of the Different Molecular Weight Fractions

**Figure 4:**
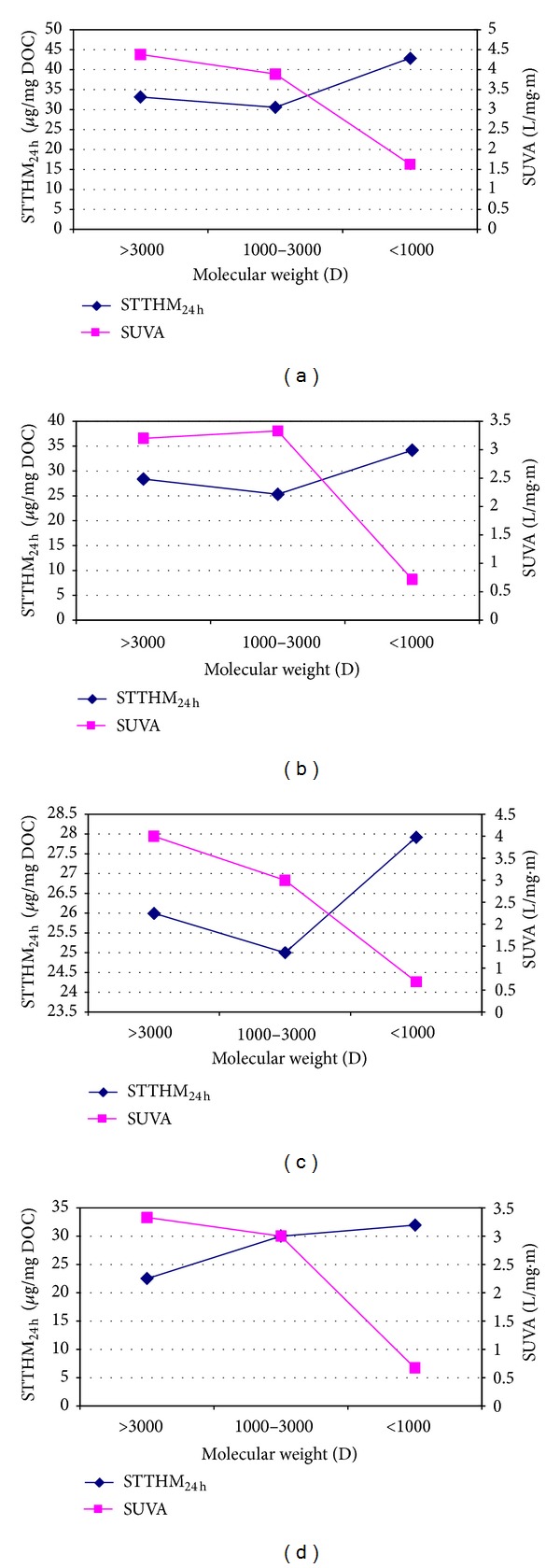
Variations of the SUVA_254_ and STTHM_24 h_ of physical fractions in the NOM for (a) raw water, (b) ozonation process, (c) coagulation-flocculation process, and (d) filtration process.

SUVA is a good surrogate parameter for understanding the humic content and a good predictor parameter for representing the aromatic carbon content of NOM in water as well. Meanwhile, it can be related to THM reactivity, described as generated TTHM per unit of DOC or specific TTHM (STTHM).  Figure 4 presents the relationships in the values of STTHM_24 h_ (generated TTHM per unit of DOC for the reaction time of 24 hours) and SUVA with regard to the AMW fractions from the different treatment stages.

STTHM_24 h_ values for NOM fractions at all water samples were 18 *μ*g-TTHM/mg-DOC to 42 *μ*g-TTHM/mg-DOC While the SUVA values were lower than 2 L/mg·m for AMW < 1 K, they were higher than 3 L/mg·m for the other fractions. Although the fraction of AMW < 1 K had the lowest SUVA value (<2 L/mg·m), it had the highest reactivity among all the fractions. Nonetheless, the lowest yield coefficient (22.5 *μ*g-TTHM/mg-DOC) was observed for the fractions with AMW greater than 3 K. This result also demonstrated that as the molecular weight of the fractions decreased, STTHM_24 h_ values increased. Similar results were obtained by some researches [21, 27] in that lower organic substances (AMW < 1 K) contributed to the most of DBPs, per unit organic carbon and per unit of chlorine oxidized.

## 4. Conclusions

In this study, the water samples collected from the treatment stages in KWTP, a main conventional treatment plant, were separated according to molecular weight cut-off using various UF membranes. The fraction with AMW < 1 K was the predominant organic matter fraction among all NOM fractions, in accordance with the results of mass balance on DOC determinations. The results of DOC concentration related to the fractions of AMW > 30 K, 30−10 K, 10−5 K, and 5−3 K were not reported because DOC measurements of these fractions were <0.1 mg/L in all water samples. The highest DOC and UV_254_ values were obtained with the fraction of AMW < 1 K among the other fractions in raw water samples, as 4.1 mg/L and 0.06 cm^−1^. One of the most important findings is that the lowest molecular weight fractions (<1 K), known as hydrophilic compounds, play a greater role in the formation of THMs. TTHM yield coefficients ranged from 18 to 42 *μ*g-TTHM/mg-DOC. Although the fraction of AMW < 1 K had the lowest SUVA values (<2 L/mg·m), it had the highest THM reactivity among all the fractions. In addition, as the molecular weight of the fractions decreased, STTHM_24 h_ values increased. This result also shows that the NOM fraction with AMW less than 1 K, consisting of hydrophilic compounds, is the major THM precursor.

The determination of NOM fractions with the UF technique may be a good alternative approach for operating conventional treatment plants with respect to applied coagulants such as alum or disinfectant dose using chlorine.

## Figures and Tables

**Table 1:** Physicochemical characteristics of Terkos raw water samples.

Parameter	Unit	Terkos Lake
pH	—	7.77
Turbidity	NTU	1.41
Total hardness	mg CaCO_3_/L	138
Alkalinity	mg CaCO_3_/L	113
Cl^−^	mg/L	23
Temperature	°C	22.3
DOC	mg/L	5.68
UV_254_	cm^−1^	0.125
Br^−^	*μ*g/L	80
THMFP	*μ*g/L	292
SUVA	L/mg·m	2.07

**Table 2:** DOC mass balance and recovery for each water treatment unit.

Water source	Molecular dimension distances	DOC	Sample volume	DOC mass	DOC distribution
	Dalton (D)	(mg/L)	(L)	(mg)	(%)
Raw water	Raw water	5.68	0.18	1.0224	
	>30000 D (30 K)	0.13	0.54	0.0702	6.23
	30000–10000 D (30–10 K)	0.12	0.54	0.0648	5.75
	10000–5000 D (10 K–5 K)	0.14	0.54	0.0756	6.71
	5000–3000 D (5 K–3 K)	0.16	0.54	0.0864	7.67
	3000–1000 D (3 K–1 K)	0.18	0.54	0.0972	8.63
	<1000 D (<1 K)	4.1	0.18	0.738	65.01
	Total mass = >30 K + 30–10 K + 10–5 K + 5–3 K + 3–1 K + <1 K			1.1268	100
	Recovery (%) = raw water/total mass				110.21

Ozonation	Ozonated water	5.53	0.18	0.9954	
	>3000 D (>3 K)	0.25	0.54	0.154286	16.46
	1000–3000 D (1 K–3 K)	0.15	0.54	0.0594	6.34
	<1000 D (<1K)	4.02	0.18	0.7236	77.20
	Total mass = >3 K + 1–3 K + <1 K			0.937286	100
	Recovery (%) = ozonated water/total mass				106.2

Coagulation-flocculation	Coagulated water	4.1	0.18	0.738	
	>3000 D (>3 K)	0.15	0.54	0.081	11.36
	1000–3000 D (1 K–3 K)	0.12	0.54	0.0648	9.09
	<1000 D (<1 K)	3.17	0.18	0.567	79.55
	Total mass = >3 K + 1–3 K + <1 K			0.7128	100
	Recovery (%) = coagulated water/total mass				103.53

Filtration	Filtrated water	3.19	0.18	0.5742	
	>3000 D (>3 K)	0.12	0.54	0.054	10.10
	1000–3000 D (1 K–3 K)	0.1	0.54	0.0216	4.04
	<1000 D (<1 K)	2.55	0.18	0.459	85.86
	Total mass = >3 K + 1–3 K + <1 K			0.5346	100
	Recovery (%) = filtrated water/total mass				107.41
